# Hippocampal 2-Arachidonoyl Glycerol Signaling Regulates Time-of-Day- and Stress-Dependent Effects on Rat Short-Term Memory

**DOI:** 10.3390/ijms21197316

**Published:** 2020-10-03

**Authors:** Alessia Santori, Maria Morena, Matthew N. Hill, Patrizia Campolongo

**Affiliations:** 1 Department of Physiology and Pharmacology, Sapienza University of Rome, 00185 Rome, Italy; alessia.santori@uniroma1.it; 2 Neurobiology of Behavior Laboratory, Santa Lucia Foundation, 00143 Rome, Italy; 3 Hotchkiss Brain Institute, University of Calgary, Calgary, AB T2N 4N1, Canada; mmorena@ucalgary.ca (M.M.); mnhill@ucalgary.ca (M.N.H.); 4 Department of Cell Biology and Anatomy & Psychiatry, University of Calgary, Calgary, AB T2N 4N1, Canada

**Keywords:** time-of-day, short-term memory, endocannabinoids, hippocampus, swim stress

## Abstract

Background: Cannabinoids induce biphasic effects on memory depending on stress levels. We previously demonstrated that different stress intensities, experienced soon after encoding, impaired rat short-term recognition memory in a time-of-day-dependent manner, and that boosting endocannabinoid anandamide (AEA) levels restored memory performance. Here, we examined if two different stress intensities and time-of-day alter hippocampal endocannabinoid tone, and whether these changes modulate short-term memory. Methods: Male Sprague-Dawley rats were subjected to an object recognition task and exposed, at two different times of the day (i.e., morning or afternoon), to low or high stress conditions, immediately after encoding. Memory retention was assessed 1 hr later. Hippocampal AEA and 2-arachidonoyl glycerol (2-AG) content and the activity of their primary degrading enzymes, fatty acid amide hydrolase (FAAH) and monoacylglycerol lipase (MAGL), were measured soon after testing. Results: Consistent with our previous findings, low stress impaired 1-hr memory performance only in the morning, whereas exposure to high stress impaired memory independently of testing time. Stress exposure decreased AEA levels independently of memory alterations. Interestingly, exposure to high stress decreased 2-AG content and, accordingly, increased MAGL activity, selectively in the afternoon. Thus, to further evaluate 2-AG’s role in the modulation of short-term recognition memory, rats were given bilateral intra-hippocampal injections of the 2-AG hydrolysis inhibitor KML29 immediately after training, then subjected to low or high stress conditions and tested 1 hr later. Conclusions: KML29 abolished the time-of-day-dependent impairing effects of stress on short-term memory, ameliorating short-term recognition memory performance.

## 1. Introduction

The impact of stress on learning and memory processes is a controversial topic that has largely been investigated to unveil its complex effects on cognition [1]. Whereas intense emotional events can generate vivid long-lasting memories [2], very strong emotional experiences can also induce amnesia [3]. The discovery of stress hormone receptors in the hippocampus has fostered research showing that this brain structure is crucially involved in the negative feedback regulation of the hypothalamic–pituitary–adrenal (HPA) axis [4]. Exposure to stress alters both hippocampal anatomy and functionality [5], with negative consequences on memory processes [6]. Indeed, the hippocampus represents a key forebrain structure highly associated with emotional and recognition memory processes [7].

Endocannabinoid signaling is widely distributed throughout corticolimbic circuits that are linked to stress response [8] and represents one of the main systems modulating hippocampal neuroplasticity [9]. The endocannabinoid system is a neuromodulatory lipid system, which consists of the cannabinoid type 1 and type 2 (CB1 and CB2) receptors [10] and two major endogenous ligands, 2-arachidonoyl glycerol (2-AG; [11]) and N-arachidonyl ethanolamide (anandamide, AEA; [12]). Endocannabinoids are retrograde messengers that are synthesized “on demand” in the postsynaptic membrane by Ca^2+^-dependent and -independent mechanisms [13] and feedback onto presynaptic terminals, thus modulating afferent neurotransmitter release via activation of CB1 receptors [14]. 2-AG and AEA are primarily degraded by distinct hydrolytic enzymes, monoacylglycerol lipase (MAGL; [15]) and fatty acid amide hydrolase (FAAH; [16]), respectively. Considerable evidence indicates that endocannabinoid signaling plays a key role in fundamental physiological processes that are altered in a circadian manner [17], simultaneously regulating both the activation of the HPA axis [18] and the termination of the stress response [19]. Stress exposure generally provokes alterations in endocannabinoid tone, depending on the intensity, duration and nature of the stressor, but also in the brain region investigated [20]. However, the interaction between stress and the endocannabinoid system [21] has typically been investigated in the same time window [22,23,24]. We previously demonstrated that stress-impairing effects on short-term recognition memory depend on the intensity of stress and time-of-day and that systemic augmentation of AEA levels restore memory performance in a stress intensity- and time-of-day-dependent fashion [25]. However, it still remains unexplored i) if different stress intensities affect hippocampal endocannabinoid system components, ii) whether the effects are time-of-day-dependent, iii) how short-term memory is influenced and iv) 2-AG’s role in such regulation. Therefore, the present study aimed to determine how different stress intensities at two times of the day (i.e., morning or afternoon) influence hippocampal endocannabinoid modulation of short-term recognition memory, and how post-training bilateral intra-CA1 infusion of the 2-AG hydrolysis inhibitor KML29 influences short-term memory performance.

## 2. Results

### 2.1. Effects of Different Stress Intensities on Hippocampal 2-AG Levels and MAGL Hydrolytic Activity in Rats Tested in the Morning or Afternoon

This experiment investigated whether different stress levels and times of the day (morning vs afternoon) associated to the test procedure induced any alteration in hippocampal 2-AG content and MAGL hydrolytic activity at the time of testing.

**Figure 1:**
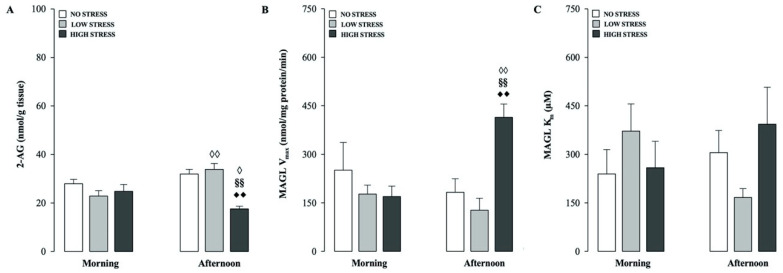
Time-of-day-dependent effects of stress on short-term recognition memory influence hippocampal 2-AG levels and its degradation. Hippocampal 2-AG levels (**A**), and MAGL V_max_ (**B**) and K_m_ (**C**) values, as assessed immediately after the testing trial in non-cannulated rats that were subjected to no, low or high stress conditions after the training trial performed in the morning or afternoon. *Post hoc* comparisons reported significant differences between groups as follows: ♦♦ *p* < 0.01 vs the corresponding no stress group. §§ *p* < 0.01 vs. the corresponding low stress group. ♢ *p* < 0.05; ♢♢ *p* < 0.01 vs the corresponding stress condition groups trained in the morning. Data are expressed as mean ± SEM (*n* = 4–10 per group).

As shown in Figure 1A, two-way ANOVA for hippocampal 2-AG levels showed a significant stress condition effect (F_(2.49)_ = 9.727, *p* = 0.0003), no significant effect of the time of testing, but a significant interaction between both factors (F_(2.49)_ = 8.559, *p* = 0.0006). *Post hoc* comparisons for hippocampal 2-AG content showed that among animals tested in the afternoon session, rats subjected to the high stress condition presented a significant decrease in 2-AG levels as compared with their corresponding no and low stress condition groups (*p* < 0.01 for both comparisons) and their morning counterpart (*p* < 0.05). Furthermore, within the low stress condition group, rats tested in the afternoon showed increased 2-AG levels relative to rats tested in the morning session (*p* < 0.01).

Figure 1B,C shows the effects of different stress intensities and times of the testing trial on hippocampal MAGL activity. Two-way ANOVA for V_max_ of MAGL reported a significant stress condition effect (F_(2.23)_ = 3.956, *p* = 0.033), no significant testing time effect and a significant interaction between both factors (F_(2.23)_ = 6.519, *p* = 0.006). *Post hoc* analysis showed a significant increase of MAGL V_max_ value in rats subjected to the high stress condition and tested in the afternoon, compared to no stress and low stress rats that were tested at the same time and to the high stress condition group tested in the morning (*p* < 0.01, for all comparisons; Figure 1B). Two-way ANOVA for MAGL K_m_ revealed no significant stress condition, testing time or stress condition × testing time interaction effects (Figure 1C).

### 2.2. Effects of Different Stress Intensities on Hippocampal AEA Levels and FAAH Hydrolytic Activity in Rats Tested in the Morning or Afternoon

This experiment investigated whether different stress levels and times of the day (morning vs afternoon) associated to the test procedures induced any alteration in hippocampal AEA content and FAAH hydrolytic activity at the time of testing.

**Figure 2:**
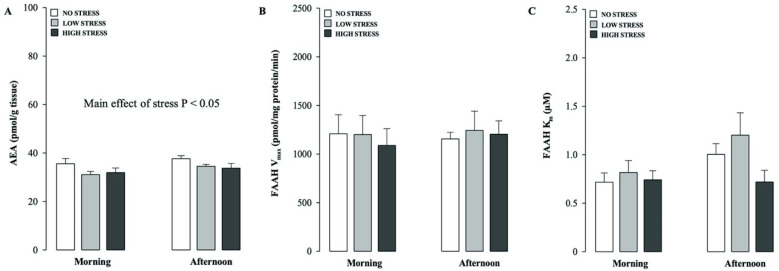
Time-of-day-dependent effects of stress on short-term recognition memory influence hippocampal AEA levels and its degradation. Hippocampal AEA levels (**A**), and FAAH V_max_ (**B**) and K_m_ (**C**) values, as assessed immediately after the testing trial in non-cannulated rats that were subjected to no, low or high stress conditions after the training trial performed in the morning or afternoon. *p* < 0.05 main effect of stress on hippocampal AEA levels. Data are expressed as mean ± SEM (*n* = 7–8 per group).

As shown in Figure 2A, two-way ANOVA for hippocampal AEA levels revealed that there was a significant stress condition effect (F_(2.49)_ = 3.388, *p* = 0.042), but no significant effect of the time of testing or interaction between both factors.

The effects of different stress intensities and times of the testing trial on hippocampal FAAH activity are shown in Figure 2B,C. Two-way ANOVAs for FAAH V_max_ or K_m_ did not reveal any significant stress condition, testing time or stress condition × testing time interaction effects (Figure 2B,C).

### 2.3. Stress Intensity and Time-of-Day Effects on Short-Term Recognition Memory

This experiment examined the effects of different stress intensities and times of the day (morning vs afternoon) on short-term memory retention performance. Each behavioral performance analyzed in the present section applies to animals treated with vehicles that were used in the subsequent KML29 experiments and it is functional to discuss the effects of different stress intensities and time-of-day on short-term recognition memory. Consistent with our previous work involving non-cannulated rats [25], here we found impairing effects of stress on short-term recognition memory, which were stress intensity- and time-of-day-dependent. All the results concerning the behavioral performance on the training trial are shown in Table 1.

**Figure 3:**
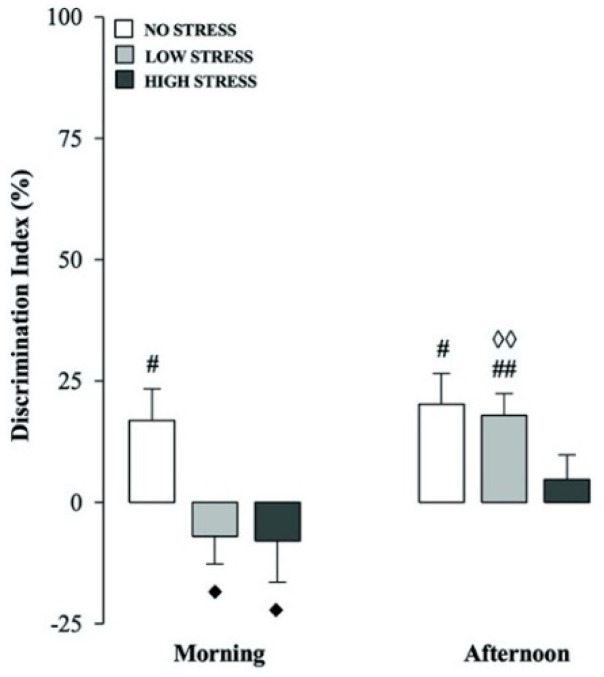
Different stress intensities and time-of-day effects on short-term memory. Discrimination index on the testing trial for intra-CA1 vehicle-treated rats belonging to the no, low or high stress condition groups that were tested in the morning or afternoon. *Post hoc* comparisons revealed significant differences between groups as follows: ♦ *p* < 0.05 vs the corresponding no stress group. ♢♢ *p* < 0.01 vs the corresponding low stress morning group. # *p* < 0.05, ## *p* < 0.01, one-sample t-tests significantly different from zero. Data are expressed as mean ± SEM (*n* = 10–12 per group).

Two-way ANOVA for discrimination index revealed significant stress condition (F_(2.63)_ = 5.517, *p* = 0.006) and time of the testing (F_(1.63)_ = 7.463, *p* = 0.008) effects, but no significant interaction between these two factors. One-sample t-tests reported that intra-CA1 vehicle-treated rats displayed discrimination indexes significantly different from zero for both the no stress condition morning and afternoon groups (t_(11)_ = 2.588, *p* = 0.025 and t_(10)_ = 3.200, *p* = 0.010, respectively; Figure 3) and only for the low stress condition group tested in the afternoon (t_(11)_ = 3.976, *p* = 0.002; Figure 3), suggesting that these experimental groups discriminated the novel object. Contrarily, rats belonging to the high stress condition groups tested either in the morning or afternoon and the low stress condition morning group did not express memory retention for the familiar object. *Post hoc* analysis indicated that exposure to the low stress condition in the morning decreased the rat discrimination index as compared to the corresponding low stress condition group tested in the afternoon (*p* < 0.01; Figure 3). Furthermore, animals belonging to both the low and high stress condition morning groups showed impaired discrimination indexes compared to the no stress condition group that was tested at the same time of the day (*p* < 0.05 for both comparisons; Figure 3). With respect to the total object exploration time on the testing trial, two-way ANOVA revealed a significant stress condition effect (F_(2.63)_ = 4.892, *p* = 0.011), but no significant time of testing or stress condition × time of testing interaction effects (Table 2). Two-way ANOVAs for number of crossings or rearings revealed a significant effect of the stress condition (F_(2.63)_ = 7.579, *p* = 0.001 and F_(2.63)_ = 17.225, *p* < 0.0001, respectively) but no time of testing or stress condition × time of testing interaction effects (Table 2).

### 2.4. Effects of the 2-AG Hydrolysis Inhibitor KML29 on Hippocampal Modulation of Short-Term Recognition Memory Performance in the No, Low and High Stress Condition Groups Tested in the Morning

This experiment investigated whether the 2-AG hydrolysis inhibitor KML29 (2 or 20 ng in 0.5 µl) bilaterally infused into the CA1 regions of the dorsal hippocampus, immediately after the training trial, modulates short-term memory performance in an object recognition task and whether these effects are influenced by exposure to different stress conditions, in the morning. Results concerning the behavioral performance on the training trial are shown in Table 1.

**Figure 4:**
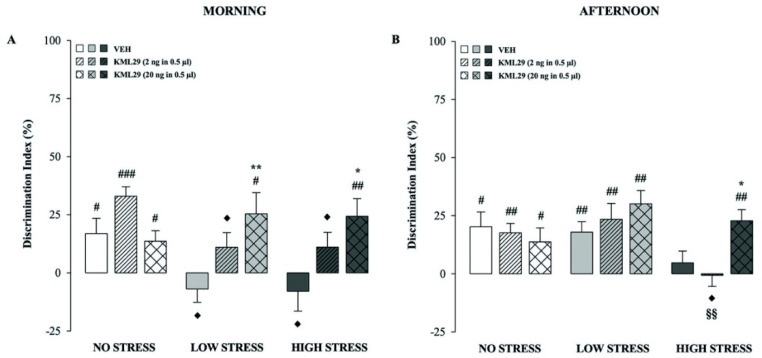
KML29 modulation of stress- and time-of-day-dependent effects on short-term memory. Discrimination index on the testing trial for rats that were administered in the CA1 region of the dorsal hippocampus with either vehicle or KML29 and then subjected to no, low or high stress conditions immediately after training, in the morning (**A**) or in the afternoon (**B**). *Post hoc* analysis reported significant group differences as follows: * *p* < 0.05, ** *p* < 0.01 vs the corresponding vehicle group. ♦ *p* < 0.05 vs the corresponding no stress group. §§ *p* < 0.01 vs the corresponding low stress group. # *p* < 0.05; ## *p* < 0.01, ### *p* < 0.0001, one-sample t-tests significantly different from zero. Data are expressed as mean ± SEM (*n* = 10–12 per group).

Two-way ANOVA for the discrimination index revealed significant stress condition (F_(2.92)_ = 3.186, *p* = 0.046), treatment (F_(2.92)_ = 8.520, *p* = 0.0004) and stress condition × treatment interaction (F_(4.92)_ = 3.134, *p* = 0.018) effects. As shown in Figure 4A, one-sample t-tests revealed that the discrimination indexes were significantly different from zero for all no stress treatment groups (t_(11)_ = 2.588, *p* = 0.025; t_(10)_ = 8.064, *p* < 0.0001 and t_(11)_ = 2.993, *p* = 0.012; vehicle, KML29 2 and 20 ng, respectively), while, for the low and high stress groups, only KML29 20 ng-treated rats discriminated the new object (t_(9)_ = 2.811, *p* = 0.020, t_(11)_ = 3.208, *p* = 0.008, for the low and high stress condition KML29 20 ng groups, respectively). In contrast, vehicle- and KML29 2 ng-treated groups in the low and high stress conditions did not express memory retention for the familiar object. *Post hoc* analysis showed that KML29 20 ng-treated rats subjected to low or high stress presented a better discrimination index relative to their corresponding vehicle groups (*p* < 0.01 and *p* < 0.05; low and high stress conditions, respectively; Figure 4A). Moreover, rats that were treated with vehicle or KML29 2 ng and then exposed to the low or high stress condition showed an impaired discrimination index compared to their corresponding no stress groups (*p* < 0.05, for all comparisons; Figure 4A). Concerning the total exploration time of the two objects on the testing trial, in accordance with our previous findings [25], two-way ANOVA revealed a significant stress condition effect (F_(2.92)_ = 12.157, *p* < 0.0001), but no significant treatment or stress condition × treatment effects (Table 2). Finally, rats’ exploratory behavior of the arena during the test trial showed significant differences among experimental groups arisen from the different stress exposures. Two-way ANOVAs for number of crossings and rearings revealed a significant stress condition effect (F_(2.92)_ = 9.387, *p* = 0.0002 and F_(2.92)_ = 42.565, *p* < 0.0001, respectively) but no treatment or stress condition × treatment interaction effects (Table 2).

### 2.5. Effects of the 2-AG Hydrolysis Inhibitor KML29 on Hippocampal Modulation of Short-Term Recognition Memory Performance in the No, Low and High Stress Condition Groups Tested in the Afternoon

This experiment investigated whether the 2-AG hydrolysis inhibitor KML29 (2 or 20 ng in 0.5 µl) bilaterally infused into the dorsal CA1, immediately after the training trial, modulates short-term memory performance in an object recognition task and whether these effects are influenced by exposure to different stress conditions, in the afternoon. Results concerning the behavioral performance on the training trial are shown in Table 1.

A two-way ANOVA for discrimination index revealed a significant stress condition (F_(2.95)_ = 5.916, *p* = 0.004) effect but no significant effect of treatment or interaction between these two factors. As shown in Figure 4B, one-sample t-tests revealed that the discrimination indexes were significantly different from zero for the no stress and low stress vehicle, KML29 2 and KML29 20 ng groups (t_(10)_ = 3.200, *p* = 0.010; t_(10)_ = 4.336, *p* = 0.002 and t_(10)_ = 2.274, *p* = 0.046, respectively, for no stress groups; t_(11)_ = 3.976, *p* = 0.002; t_(10)_ = 3.446, *p* = 0.006 and t_(11)_ = 5.258, *p* = 0.0003, respectively, for low stress condition groups) and the high stress condition KML29 20 ng group (t_(11)_ = 4.765, *p* = 0.0006), indicating that these animals were capable to discriminate the novel object. The remaining rats that were subjected to the high stress condition and administered with either vehicle or KML29 2 ng did not express memory retention for the familiar object (Figure 4B). *Post hoc* comparisons showed that, among rats tested under the high stress condition, KML29 20 ng significantly increased the discrimination index as compared to animals that were treated with vehicle (*p* < 0.05; Figure 4B), whereas KML29 2 ng impaired rat discrimination index in comparison to their corresponding no stress and low stress groups (*p* < 0.05 and *p* < 0.01, respectively; Figure 4B). Consistent with our previous findings [25], a two-way ANOVA for the total exploration time of the two objects on the testing trial revealed a significant effect of stress condition (F_(2.95)_ = 4.116, *p* = 0.019) but no significant treatment or stress condition × treatment effects (Table 2). A two-way ANOVA for number of crossings and rearings revealed a significant effect of stress condition (F_(2.95)_ = 9.065, *p* = 0.0002 and F_(2.95)_ = 11.190, *p* < 0.0001, respectively) but no significant treatment or stress condition × treatment interaction effects (Table 2).

## 3. Discussion

The present findings show that different stress intensities and times of day differentially modulate hippocampal endocannabinoid tone. Exposure to high stress impairs short-term recognition memory selectively before the onset of the activity phase (afternoon), but not during the inactive phase (morning), and decreases hippocampal 2-AG content, presumably by increasing MAGL hydrolytic activity. Our results indicate that boosting hippocampal 2-AG signaling, with post-training bilateral intra-CA1 injections of the 2-AG hydrolysis inhibitor KML29, completely restores impaired memory performance, in accordance to the stress intensity and phase activity/inactivity.

Evidence has demonstrated the abundant expression of CB1 receptors within cortico-limbic regions, including the basolateral complex of the amygdala (BLA), hypothalamus, hippocampus and medial prefrontal cortex (mPFC) [26,27]. CB1 receptors activation regulates the HPA axis activity [20] but also stress and emotional arousal effects on memory [28]. It has been shown that exposure to stress activates corticotropin-releasing hormone (CRH) receptors 1 (CRHR1) in the amygdala, which increase the enzymatic activity of FAAH, resulting in a decrease of the inhibitory tone of AEA. Such a mechanism contributes to the activation of the HPA axis and stress-related behavioral responses [20]. Conversely, elevations in corticosterone appear to be the primary mechanism by which stress increases 2-AG levels in the hypothalamus, which activating CB1 receptors contributes to negative-feedback inhibition of the HPA axis and termination of stress response [20].

The effects of stress on the endocannabinoid system are complex, regionally specific and time-dependent [20]. Several studies demonstrated that exposure to acute stress generally causes a rapid reduction in AEA content in response to an array of stressors [29,30], whereas it typically increases 2-AG signaling throughout different cortico-limbic regions [26,31], suggesting a bidirectional effect of stress on the endocannabinoid system. Specifically, within the hippocampus, acute restraint stress reduces AEA content and increases 2-AG levels [32,33]. In line with this evidence, our findings show that independent of the time of the day and stress intensity, swim stress decreased hippocampal AEA levels. Concerning 2-AG’s tone, however, we did not find any increase in hippocampal 2-AG immediately after acute stress exposure, as most of the studies in literature have normally documented. Indeed, we found a strong reduction of 2-AG content, along with a robust increase of the activity of its degrading enzyme, in rats exposed to the high stress condition and tested at the onset of the active phase.

According to the timing of stress exposure, stress-mediated secretion of glucocorticoids alters hippocampal functions and plasticity [34], thus affecting hippocampal-dependent memories in rodents and humans [35].

It is now well established that exposure to glucocorticoids, a stressor or emotional arousal, shortly before, during or immediately after training, impairs short-term memory performances in an object recognition task [25,36], likely by negatively interfering with memory retrieval [37]. Since corticosterone is still elevated at the time of the 1-h retention test, it is probable that it affected short-term retention performance via direct influences on the retrieval of memory processing [38].

By replicating our previously published findings [25], here we found that when animals were tested during the circadian low activity phase of the HPA axis (i.e., morning session), exposure to a stressor, regardless of its intensity, impaired memory performance. Conversely, when testing occurred at the beginning of their active phase (i.e., afternoon), when the HPA axis reaches its activity peak [39], under laboratory conditions of a regular light/dark cycle, only the high intensity stressor impaired memory performance.

Similar to corticosterone, activity of arousal system mediators is also influenced by circadian rhythm, with norepinephrine reaching its peak at the onset of the dark phase [40].

In the light of this evidence, as we had previously speculated [25], exposure to stress impairs memory retention only when it causes a more robust deviation from homeostasis, that is during the low activity phase of stress systems. Thus, it is likely that exposure to the low intensity stressor at the beginning of the active phase did not alter behavioral performance, because it did not cause a severe deviation from homeostasis, with animals tested during their high HPA axis and arousal system activity phase.

Our surprising finding that only exposure to a high stress in the afternoon, but not in the morning, induces a strong reduction of hippocampal 2-AG levels might be the result of a compensatory and still unresolved mechanism that allows the system at the onset of the dark phase, which, thus, already presents high hippocampal corticosterone and norepinephrine levels, to perceive and initiate a proper stress response, by reducing both 2-AG inhibitory action at hippocampal noradrenergic fibers and its negative feedback regulation onto the HPA axis. Conversely, exposure to a lower intensity stressor might have been not strong enough to activate this putative mechanism. This hypothesis is also supported by corticosterone plasma levels of the low stress exposure group tested in the afternoon, which did not differ from those of non-stressed controls [25].

It is important to note that studies examining the effects of stress on endocannabinoid content have often been performed ignoring that the timing of the experiments could influence stress modulation of the endocannabinoid system and memory processes. Thus, to our knowledge this is the first study documenting an interaction between stress exposure and time-of-day on hippocampal 2-AG levels, and this might explain the contrasting findings in literature concerning the endocannabinoid, and particularly 2-AG, modulation of memory. Further investigation is warranted to explore our novel findings.

We previously reported that systemic post-training injections of the AEA hydrolysis inhibitor URB597, which increases AEA levels, counteracted detrimental effects of stress on short-term memory, likely by restoring corticosterone to physiological levels, when altered by swim stress exposure. Our results highlight that URB597-mediated beneficial effects on memory are not hippocampus-dependent, as we found a consistent reduction of hippocampal AEA levels induced by stress in general. Evidence examining local manipulation of endocannabinoid signaling in the BLA has consistently found that increased AEA signaling is essential for enhancing the consolidation of emotional memories [41,42], making the BLA a possible candidate for AEA modulation of stress effects on memory [24]. Simultaneously, since different contributions of the perirhinal, prefrontal and parahippocampal cortexes have been documented in memory processes [43], such brain areas could account for AEA beneficial effects on stress-induced alteration of recognition memory.

Extensive evidence demonstrated that glucocorticoids, through a rapid non-genomic mechanism, recruit 2-AG signaling within the hippocampus to impair memory retrieval of fear memories [22,38] through downstream activation of the hippocampal noradrenergic system [20]. Our results show that intra-CA1 administration of the 2-AG hydrolysis inhibitor KML29 counteracts the detrimental effects of stress on memory. It should be noted, however, that the studies mentioned above evaluated the interaction between stress and hippocampal 2-AG in types of memory and behavioral tasks different from those employed in our current study. Future studies will evaluate whether stress intensities, time-of-day and endocannabinoid tone also affect cortical (e.g., parahippocampal or perirhinal) modulation of recognition memory. Literature data suggested that recognition memory reflects the contribution of recollection and familiarity as two separable memory retrieval processes, indicating the hippocampus and the parahippocampal cortex as brain regions crucial for recollection, whereas the perirhinal cortex is necessary for familiarity-based recognition [44,45]. The present paper focused on hippocampal modulation of memory because i) compelling evidence demonstrated that the dorsal hippocampus is critical for object recognition memory [46]; ii) it has been repeatedly demonstrated that hippocampal vulnerability and sensitivity to stress affects memory and neuroplasticity [34] and iii) endocannabinoids in the hippocampus crucially modulate stress effects on memory (i.e., short-term memory) [47].

Interestingly, it has been shown that activation of CB1 receptors on adrenergic and noradrenergic cells reduces the release of adrenaline and noradrenaline at both the peripheral and central level [48,49]. Besides the interaction with the arousal noradrenergic system, several studies report a mutual regulation between glucocorticoids and endocannabinoids, where glucocorticoids influence the endocannabinoid response, which in turn, modulates glucocorticoid secretion through local and distal regulation of HPA axis activity [50].

Specifically, while the neuropeptide CRH, rapidly released in response to stress [51,52], reduces AEA signaling at glutamatergic neurons, which probably contributes to HPA axis activation [29], glucocorticoids enhance 2-AG’s synthesis [19,53] to terminate the stress response throughout the HPA axis negative feedback regulation in limbic brain regions [26].

Collectively, these data seem to suggest that hippocampal 2-AG signaling might be responsible for the regulation of noradrenergic release, by exerting inhibitory control over noradrenergic fibers, and participate to the negative feedback regulation of the HPA axis. Supporting this hypothesis, the hippocampus represents an important site of negative feedback regulation of the HPA axis activity [54].

Therefore, it is tentative to speculate that our intervention might have reduced hippocampal norepinephrine release, which impairs memory retrieval [38], facilitating negative feedback on the HPA axis with a faster recovery from stress, and, ultimately, restored memory performance, highlighting that MAGL inhibition might be a potential therapeutic target for treating stress-induced memory performance deficits.

## 4. Materials and Methods

### 4.1. Animal Care and Use

Male adult Sprague-Dawley rats (10 weeks of age; 350–380 g at the time of behavioral experiments, Charles River Laboratories, Calco, Italy) were single housed in a temperature-controlled (21 ± 1 °C) colony room and maintained under a 12 h/12 h light/dark cycle (07:00 am to 7:00 pm lights on). Food and water were available ad libitum. All behavioral procedures were performed during the light phase of the cycle between 10:00 am and 6:00 pm. All experimental procedures were performed in compliance with the ARRIVE guidelines, the European Union Directive on the protection of animals used for scientific purposes (2010/63/EU) and the D.L. 26/2014 of Italian Ministry of Health.

### 4.2. Surgery

Rats were anesthetized with ketamine hydrochloride (100 mg kg^−1^) and xylazine (7 mg kg^−1^), given intraperitoneally (i.p.). Successively, animals were subcutaneously injected with saline (3 ml) to facilitate clearance of drugs and prevent dehydration. Rats were then placed in a stereotaxic frame (David Kopf Instruments, Tujunga, CA, USA), and two 23-gauge (11-mm-long) stainless-steel guide cannulae were implanted bilaterally 2 mm above the CA1 region of the dorsal hippocampus (AP, −3.3 mm; ML, ±1.7 mm; DV, −2.7 mm) [22,38,41]. The cannulae were affixed to the skull with two anchoring screws and dental cement. Stylets (11-mm-long 00 insect dissection pins) were inserted into each cannula to prevent clogging. After surgery, rats were retained on a heated pad to recover from anesthesia and were then returned to the home cage. Rats were allowed to recover from surgery for two weeks before testing.

### 4.3. Drug Administration

The 2-AG hydrolysis inhibitor KML29 (1,1,1,3,3,3-Hexafluoropropan-2-yl 4-[bis(1,3-benzodioxol-5-yl)-hydroxymethyl]piperidine-1-carboxylate) (2 ng or 20 ng in 0.5 µl per side; Tocris Bioscience, Bristol UK) or its vehicle were bilaterally infused into the CA1 region of the hippocampus immediately after the training trial and right before the swim stress procedure, in order to block any possible stress-induced increase in 2-AG hydrolyzation. Doses were selected on the basis of previous published papers and pilot experiments performed in our laboratory [23,55]. All drugs were dissolved in 5% polyethylene glycol, 5% Tween-80, and 90% saline (vol/vol). Post-training bilateral infusions of drugs or an equivalent volume of vehicle into the CA1 were made by using a 30-gauge injection needle connected by polyethylene tubing (PE-20) to a 10 ml Hamilton microsyringe driven by a minipump (KD Instruments, Canning Vale, Australia) over a period of 50 s [41]. The injection needles protruded 2 mm beyond each cannula tip and were retained within the cannulae for an additional 20 s after drug infusion to maximize diffusion and to prevent drug backflow into the cannulae. All drug solutions were freshly prepared before each experiment.

### 4.4. Behavioral Procedures

**Figure 5:**
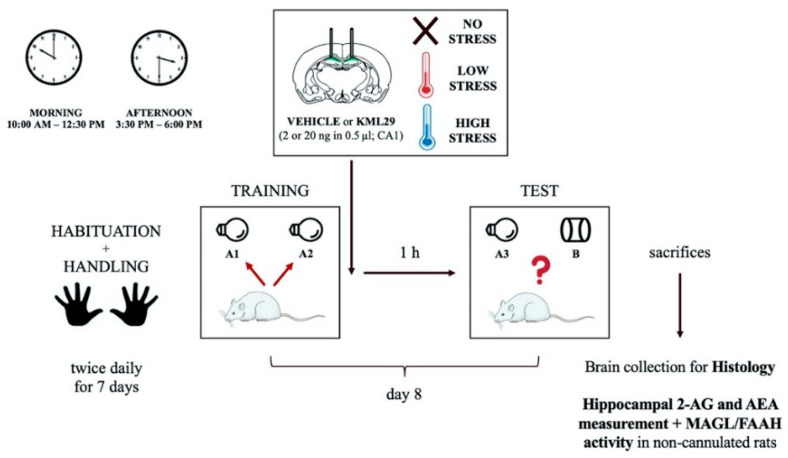
Diagram of the experimental procedures.

Object recognition task. A previously validated procedure described by [25] was used. All animals were randomly assigned to the no, low or high stress condition groups and tested either during rats’ inactive phase (morning, 10:00 am–12:30 pm) or before the onset of the activity phase (afternoon, 3:30 pm–6:00 pm). On the training trial, each rat was individually placed in the object recognition arena at the opposite end from the two identical objects. Memory retention was tested 1 h after training. On the testing trial, one copy of the familiar object (A3) and a new object (B) were placed in the same location as stimuli during the training trial (Figure 5). To reduce potential biases due to preference for particular locations or objects, all combinations and locations of objects were used. Cognitive performance during the testing trial was assessed by calculating a discrimination index as the difference in time exploring the novel and the familiar object, expressed as the percentage ratio of the total time spent exploring both objects. See Supplementary Materials for additional details.

Swim stress procedure. Swim stress was used because its neurochemical and hormonal effects are well defined and meet the criteria of a stress-inducing agent [56]. Immediately after the training trial of the object recognition task, rats were forced to swim in a tank (50 cm in height × 20 cm in diameter), filled to a depth of 30 cm with water, in a separate room from the one where the object recognition task was performed. Subsequently, rats were removed from the tank and gently wiped to dryness with absorbent paper before returning to the home cage. Rats belonging to the low and high stress condition groups were subjected to a 1- or 5-min swim stress procedure at different water temperatures of 25 ± 1 °C or 19 ± 1 °C, respectively, known to elicit different plasma corticosterone levels [22], as we recently reported using the same behavioral procedure [25].

### 4.5. Endocannabinoid Extraction and Analysis

In a cohort of animals which did not receive cannulation surgery, hippocampal 2-AG and AEA content was measured in rats belonging to the no, low and high stress condition groups that were sacrificed immediately after the testing trial, in the morning or afternoon. After rapid decapitation, hippocampi were rapidly dissected, frozen on dry ice and stored at –80 °C. The lipid extraction process and analysis of 2-AG and AEA were performed as previously described [22,55] and are detailed in the Supplementary Materials.

### 4.6. Membrane Preparation

To measure MAGL and FAAH activity, immediately after the testing trial, following rapid decapitation, the hippocampi were dissected from non-cannulated rats that were subjected to no stress, low stress or high stress conditions in the morning or afternoon. Brains were stored at –80 °C. Membrane samples were collected by homogenization of frozen tissue in TME buffer (50 mM Tris HCl, pH 7.4; 1 mM EDTA, and 3 mM MgCl_2_; 10 volumes) [22,57]. Successively, homogenates were centrifuged at 18,000× *g* for 20 min, and the resulting crude membrane fraction-containing pellet was resuspended in 10 volumes of TME buffer. To determine protein concentrations, the Bradford method (Bio-Rad) was used. Membranes were then used for MAGL and FAAH activity assays.

### 4.7. MAGL Activity Assay

MAGL activity was measured by conversion of 2-oleoylglycerol labeled with [^3^H] ([^3^H] 2-OG) in the glycerol portion of the molecule to [^3^H] glycerol preparations [22]. A slightly modified procedure of that described by [58] was used. See Supplementary Materials for additional details.

### 4.8. FAAH Activity Assay

FAAH activity from hippocampal membranes was measured by conversion of AEA labeled with [^3^H] in the ethanolamine portion of the molecule to [^3^H] ethanolamine preparations, as reported previously [22] (see Supplementary Materials).

### 4.9. Histology

Cannulated rats were anesthetized with an overdose of ketamine hydrochloride (120 mg kg^−1^, i.p.) and xylazine (20 mg kg^−1^, i.p.) and perfused transcardially with 0.9% saline. Brains were removed and stored at room temperature in 4% paraformaldehyde solution for a minimum of 24 h, followed by storage in a 20% sucrose solution in saline for cryoprotection for additional 24–48 h before sectioning. Coronal sections of 40 μm were collected on a cryostat, mounted on gelatin-coated slides, and stained with cresyl violet. Brain sections were examined under a light microscope (Nikon 801 Microscope, Italy) and the location of infusion needle tips in the CA1 of the dorsal hippocampus were made according to the standardized atlas plates of [59] by an observer blind to drug treatment condition. For all experiments, only rats with infusion needle tips within the boundaries of the targeted brain region were included in the data analysis. Approximately 15% of the animals were excluded because of either cannula misplacement or damage to the targeted tissue.

### 4.10. Data and Statistical Analysis

Object recognition data, hippocampal endocannabinoid content and MAGL and FAAH activity parameters were analyzed by two-way ANOVAs. One-sample t-tests were used to determine whether the discrimination index was different from zero. Tukey–Kramer *post hoc* test was performed to control for significant differences between groups when appropriate. Significance was considered for *p* < 0.05. Prior findings indicate that only rats that reached a minimum criterion of total object exploration time > 10 s on either the training or testing trial adequately acquire the task and can be included in the statistical analysis [25,60]. Each measure is expressed as mean ± standard error of the mean (SEM).

## Figures and Tables

**Table 1:** Exploratory behavior on the training trial for vehicle- and KML29-treated rats that were subjected to no, low or high stress conditions immediately after the training trial, in the morning or afternoon.

		Morning			Afternoon	
	Total Object Exploration Time	Number of Crossings	Number of Rearings	Total Object Exploration Time	Number of Crossings	Number of Rearings
**NO STRESS**						
VEHICLE	59.8 ± 3.5	45.5 ± 1.6	44.8 ± 4.2	50.4 ± 4.0	43.0 ± 3.3	32.0 ± 2.2
KML 2 ng	66.7 ± 3.9	52.4 ± 3.4	39.4 ± 2.9	54.2 ± 3.5	48.0 ± 3.4	41.4 ± 3.2
KML 20 ng	61.0 ± 3.5	48.8 ± 1.8	45.8 ± 2.8	58.8 ± 3.8	43.5 ± 3.9	37.0 ± 2.5
**LOW STRESS**						
VEHICLE	80.0 ± 11.7	51.1 ± 2.8	52.0 ± 4.8	53.1 ± 3.0	42.5 ± 2.8	35.2 ± 2.5
KML 2 ng	67.5 ± 8.1	59.2 ± 1.4	49.2 ± 3.9	56.9 ± 3.5	42.0 ± 2.1	37.6 ± 3.3
KML 20 ng	62.7 ± 6.6	49.1 ± 3.2	47.8 ± 3.7	54.2 ± 2.8	46.0 ± 2.2	37.1 ± 2.2
**HIGH STRESS**						
VEHICLE	62.8 ± 7.6	52.5 ± 4.7	46.3 ± 5.0	56.9 ± 3.3	46.1 ± 2.9	40.3 ± 2.8
KML 2 ng	62.3 ± 7.0	51.0 ± 3.6	43.1 ± 4.3	66.3 ± 5.5	48.8 ± 2.9	40.9 ± 2.3
KML 20 ng	63.6 ± 5.8	49.5 ± 2.6	39.8 ± 2.6	53.0 ± 3.1	48.2 ± 4.1	40.7 ± 3.3

Total time spent exploring the two objects (in seconds, s) and the number of crossings and rearings of all groups tested in the morning and in the afternoon. Data are expressed as mean ± SEM (*n* = 10–12 per group).

**Table 2:** Exploratory behavior on the testing trial for vehicle- and KML29-treated rats that were subjected to no, low or high stress conditions immediately after the training trial, in the morning or afternoon.

		Morning			Afternoon	
	Total Object Exploration Time	Number of Crossings	Number of Rearings	Total Object Exploration Time	Number of Crossings	Number of Rearings
**NO STRESS**						
VEHICLE	45.1 ± 3.4	20.7 ± 2.7	37.4 ± 3.5	40.6 ± 4.5	26.6 ± 3.9	33.6 ± 3.7
KML 2 ng	48.7 ± 4.6	19.2 ± 2.6	33.7 ± 3.1	29.7 ± 3.4	20.1 ± 3.7	29.1 ± 4.1
KML 20 ng	50.6 ± 4.1	22.3 ± 2.9	34.6 ± 3.4	42.3 ± 4.0	20.5 ± 3.3	32.6 ± 3.5
**LOW STRESS**						
VEHICLE	25.8 ± 3.1 **	13.1 ± 2.8 *	15.2 ± 2.1 **	32.6 ± 3.8	12.6 ± 2.1 *	24.6 ± 3.8
KML 2 ng	34.7 ± 3.7	14.1 ± 2.4	20.4 ± 2.8 **	33.7 ± 4.2	11.6 ± 2.5	21.7 ± 3.1
KML 20 ng	29.3 ± 3.1 *	12.4 ± 2.8 *	20.1 ± 3.0 **	33.3 ± 4.3	13.0 ± 2.5	18.3 ± 3.2 *
**HIGH STRESS**						
VEHICLE	36.7 ± 4.4	19.0 ± 2.4	16.8 ± 3.4 **	26.2 ± 4.3	15.4 ± 3.5	16.0 ± 3.3 **
KML 2 ng	34.9 ± 4.5	20.5 ± 2.8	18.4 ± 2.5 **	22.9 ± 3.0	13.7 ± 2.4	19.2 ± 3.3
KML 20 ng	28.1 ± 4.3 **	17.0 ± 2.9	14.2 ± 2.6 **	27.4 ± 5.2	16.2 ± 3.1	17.5 ± 3.8 *

Total time spent exploring the two objects (in seconds, s) and the number of crossings and rearings of all groups tested in the morning and in the afternoon. * *p* < 0.05; ** *p* < 0.01 vs the corresponding no stress group. Data are expressed as mean ± SEM (*n* = 10–12 per group).

## Supplementary Materials

Supplementary materials can be found at https://www.mdpi.com/1422-0067/21/19/7316/s1.

## Abbreviations

2-AG	2-arachidonoyl glycerol
AEA	Anandamide
ANOVA	Analysis of variance
BLA	Basolateral complex of the amygdala
CB1	Cannabinoid type-1
CB2	Cannabinoid type-2
CRH	Corticotropin-releasing hormone
CRHR1	Corticotropin-releasing hormone receptors 1
FAAH	Fatty acid amide hydrolase
HPA	Hypothalamic–pituitary–adrenal axis
KML29	1,1,1,3,3,3-Hexafluoropropan-2-yl 4-[bis(1,3-benzodioxol-5-yl)-hydroxymethyl]piperidine-1- carboxylate
MAGL	Monoacylglycerol lipase
mPFC	Medial prefrontal cortex
SEM	Standard error of the mean
