# Supplementary material for: Hippocampal 2-Arachidonoyl Glycerol Signaling Regulates Time-of-Day- and Stress-Dependent Effects on Rat Short-Term Memory
Source: Int J Mol Sci. 2020 Oct 3;21(19):7316. doi: 10.3390/ijms21197316 (PMC7582511; doi:10.3390/ijms21197316)
Supplement: Supplementary file 1 [file ijms-21-07316-s001.pdf]

6. Supplementary Materials

6.1. Results

6.1.1. Different stress intensities and time-of-day effects on short-term recognition memory retention performance

Training trial. Two-way ANOVA for total exploration time of the two identical objects on the training trial, before stress exposure, revealed no significant post-training stress condition effect, no significant effect of the time of training or an interaction between both factors (**Table S1**). Examination of rats' exploratory behavior of the experimental apparatus during the training trial indicated that there were no significant differences (**Table S1**) between groups. Specifically, two-way ANOVAs for number of crossings or rearings on the training trial revealed no significant post-training stress condition, time of the trial or post-training stress condition × time of the trial effects.

6.1.2. Effects of the 2-AG hydrolysis inhibitor KML29 on hippocampal modulation of short-term object recognition memory performance in the no, low and high stress condition groups tested in the morning

Training trial. Two-way ANOVA for total exploration time of the two identical objects on the training trial, before drug administration and stress exposure, revealed no significant post-training treatment effect, no significant post-training stress condition effect and no interaction between both factors (**Table S1**). Examination of rats' exploratory behavior of the experimental apparatus during the training trial revealed no significant differences for crossings and rearings among groups before drug treatment and stress exposure (**Table S1**). Two-way ANOVAs for number of crossings or rearings on the training trial revealed no significant post-training treatment effect, post-training stress effect and no significant post-training stress condition × treatment interaction effects.

6.1.3. Effects of the 2-AG hydrolysis inhibitor KML29 on hippocampal modulation of short-term object recognition memory performance in the no, low and high stress condition groups tested in the afternoon

Training trial. Two-way ANOVA for total object exploration time on the training trial, before drug administration and stress exposure, revealed no significant post-training treatment, post-training stress condition or post-training treatment × stress condition interaction effects (**Table S1**). Examination of rats' exploratory behavior of the experimental apparatus during the training trial indicated that there were no significant differences in terms of crossings and rearings (**Table S1**). In fact, two-way ANOVAs for number of crossings or rearings on the training trial revealed no post-training treatment, post-training stress condition or post-training treatment × stress condition interaction effects.

6.2. Methods

6.2.1. Behavioral Procedures

Object recognition task. The experimental apparatus was a gray open-field box (in cm, 40 wide × 40 deep × 40 high) with the floor covered with sawdust, positioned in a dimly illuminated room. The objects to be discriminated were transparent glass vials (5.5 cm diameter and 5 cm height) and white glass light bulbs (6 cm diameter and 11 cm length). All rats were handled twice per day for 1 min each and extensively habituated to the experimental context twice per day for 3 min each for 7 days preceding the training day. During habituation, rats were allowed to freely explore the apparatus in the absence of objects. On the training trial, each rat was individually placed in the experimental apparatus at the opposite end from the objects. Rats were allowed to explore two

identical objects (A1 and A2) for 6 min, then were removed from the apparatus and, after drug treatment, if belonging to the low or high stress condition group, were subjected to a swim stress procedure; then, they were returned to the home cage. The no stress group was placed back to its home cage immediately after drug infusion. To avoid the presence of olfactory trails, sawdust was stirred, foecal boli were removed and the objects were cleaned with 70% ethanol after each trial. A rat's behavior was recorded by using a video camera positioned above the experimental apparatus and videos were analyzed with Observer XT 12 (Noldus Information Technology BV, Wageningen, The Netherlands) by a trained observer who was unaware of the treatment condition. Exploration of an object was defined as pointing the nose to the object at a distance of < 1 cm and/or touching it with the nose. Turning around or sitting on an object was not considered as exploration. During the training trial, the time spent exploring the two objects (total object exploration time, s) was taken as a measure of object exploration, and exploratory behavior of the experimental apparatus was analyzed by the measuring of total number of crossings and rearings. For crossings, the floor of the apparatus was divided into four imaginary squares and the total number of crossings between squares was determined. Memory retention was tested 1 h after the training trial. On the testing trial, one copy of the familiar object (A3) and a new object (B) was placed in the same location as stimuli during the training trial (Figure 1). All combinations and locations of objects were used to reduce potential biases due to preference for particular locations or objects. Each rat was placed in the apparatus for 6 min, and behavior was recorded. To analyze cognitive performance, during the test, a discrimination index was calculated as the difference in time exploring the novel and the familiar object, expressed as the percentage ratio of the total time spent exploring both objects.

6.2.2. Endocannabinoid extraction and analysis

The lipid extraction process was performed as previously detailed [22,55]. Brain tissue was weighed and placed into borosilicate glass culture tubes containing 2 ml of acetonitrile with 5 nmol of [$^2H_8$] 2-AG and 5 pmol of [$^2H_8$] AEA for extraction and homogenized with a glass rod. Tissue was sonicated for 30 min on ice water and incubated overnight at –20°C to precipitate proteins, then centrifuged at 1500 × g to remove particulates. The supernatants were transferred to a new glass tube and evaporated to dryness under N_2 gas. The samples were reconstituted in 300 μl of acetonitrile and dried again under N_2 gas. Lipid extracts were suspended in 20 μl of acetonitrile and stored at –80°C until analysis. Analysis of 2-AG and AEA was performed by liquid chromatography mass spectrometry analysis as previously detailed [22,55].

6.2.3. MAGL Activity Assay

Membranes were incubated in a final volume of 0.5 ml TME buffer (50 mM Tris-HCl, 3.0 mM $MgCl_2$, 1.0 mM EDTA, and 300 nM URB597, pH 7.4) that contained 1.0 mg ml^{-1} fatty acid-free BSA and 100,000 dpm [^3H] 2-OG. Isotherms were constructed using six concentrations of 2-OG at concentrations between 10 and 500 μM. Incubation was performed at 30°C, and the enzymatic reaction was stopped by the addition of 2 ml of chloroform/methanol (1:2). After remaining at room temperature for 30 min with frequent mixing, 0.67 ml of chloroform and 0.6 ml of water were added, and the aqueous and organic phases were separated by centrifugation at 1000 rpm for 10 min. The amount of [^3H] in 0.5 ml of each of the aqueous phases was determined by liquid scintillation counting and conversion of [^3H] 2-OG to [^3H] glycerol was calculated. The binding affinity (K_m) and maximal hydrolytic activity (V_{max}) values for this conversion were determined by fitting the data to a single-site Michaelis–Menten equation using GraphPad Prism (GraphPad Software, Inc., La Jolla, CA, USA).

6.2.4. FAAH Activity Assay

Membranes were incubated in a final volume of 0.7 ml TME buffer (50 mM Tris-HCl, 3.0 mM $MgCl_2$, 1.0 mM EDTA, pH 7.4) that contained 1.0 mg ml^{-1} fatty acid-free bovine serum albumin (BSA) and 0.2 nM [^3H] AEA. Isotherms were constructed using eight concentrations of AEA at

concentrations between 0 and 1.5 μM. Incubation was performed at 37°C, and the enzymatic reaction was stopped by the addition of 2 ml of chloroform/methanol (1:2). After remaining at room temperature for 30 min with frequent mixing, 0.67 ml of chloroform and 0.6 ml of distilled water were added, and the aqueous phases were separated by centrifugation at 1000 rpm for 10 min. The amount of [^{3}H] in 0.5 ml of each of the aqueous and organic phases was determined by liquid scintillation counting and conversion of [^{3}H] AEA to [^{3}H] ethanolamine was calculated. The K_m of AEA for FAAH and V_{max} of FAAH for this conversion were determined by fitting the data to a single-site Michaelis–Menten equation using GraphPad Prism.

6.2.5. Data and Statistical Analysis

A between-subjects experimental design was used. Data were analyzed by two-way ANOVAs. Tukey–Kramer post hoc tests were used to determine the source of the detected significances. P values of < 0.05 were considered statistically significant. All data are expressed as mean ± SEM.
